# Slow-slip events in semi-brittle serpentinite fault zones

**DOI:** 10.1038/s41598-018-24637-z

**Published:** 2018-04-18

**Authors:** A. Goswami, S. Barbot

**Affiliations:** 1 1200 E California Blvd, Pasadena, CA 91125 USA; 2 50 Nanyang Ave, Block N2-01a-15, Nanyang, 639798 Singapore

## Abstract

Slow-slip events are earthquake-like events only with much lower slip rates. While peak coseismic velocities can reach tens of meters per second, slow-slip is on the order of 10^−7±2^ m/s and may last for days to weeks. Under the rate-and-state model of fault friction, slow-slip is produced only when the asperity size is commensurate with the critical nucleation size, a function of frictional properties. However, it is unlikely that all subduction zones embody the same frictional properties. In addition to friction, plastic flow of antigorite-rich serpentinite may significantly influence the dynamics of fault slip near the mantle wedge corner. Here, we show that the range of frictional parameters that generate slow slip is widened in the presence of a serpentinized layer along the subduction plate interface. We observe increased stability and damping of fast ruptures in a semi-brittle fault zone governed by both brittle and viscoelastic constitutive response. The rate of viscous serpentinite flow, governed by dislocation creep, is enhanced by high ambient temperatures. When effective viscosity is taken to be dynamic, long-term slow slip events spontaneously emerge. Integration of rheology, thermal effects, and other microphysical processes with rate-and-state friction may yield further insight into the phenomenology of slow slip.

## Introduction

**Figure 1:**
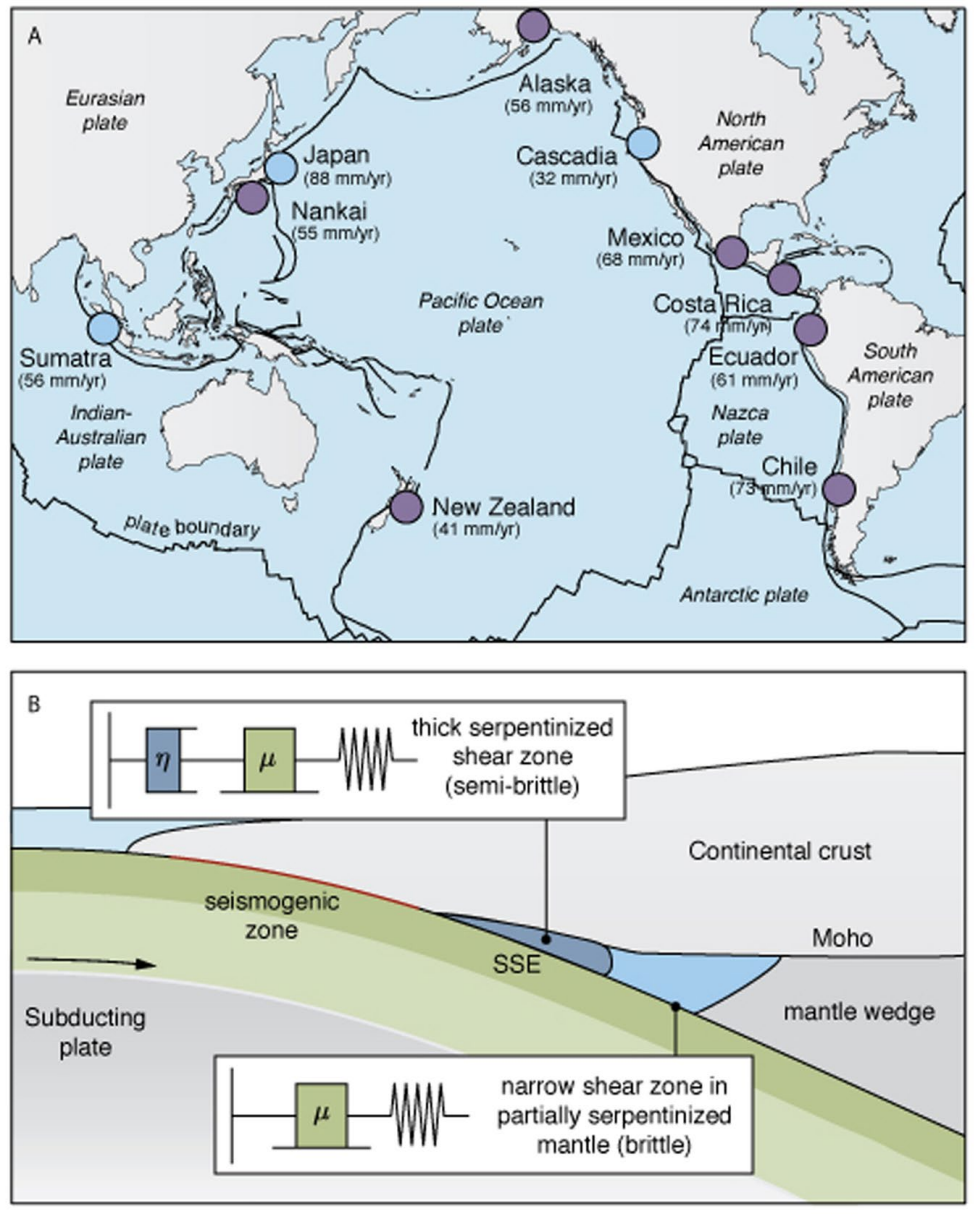
(**A**) Distribution of long-term (purple circles) and short-term (blue circles) slow-slip events detected with geodetic or paleo-geodetic measurement around the Ring of Fire (see references in the main text) and local rate of plate convergence. (**B**) Schematic cross section of a subduction zone where the megathrust near the corner of the mantle wedge below the continental Moho is deforming by a combination of brittle (localized) and viscoelastic (distributed) processes in a highly foliated serpentinized layer. Downdip the deformation is localized again and represents frictional sliding. The brittle deformation is idealized by a spring-slider constitutive model while the semi-brittle deformation is captured by a spring-slider-dashpot model (insets). (**A**) was created with GMT^96^.

The dynamics of fault slip at subduction zones can assume a wide range of behaviors. A continuum of slip modes that includes fast earthquakes, slow-slip events, and stable creep determines seismic hazards^1,2^. Along the megathrust, earthquakes, very low frequency earthquakes, and tsunami earthquakes are typically found updip of the continental Mohorovicic (Moho) discontinuity. In contrast, slow-slip events are often found close to or below the continental Moho depth at many subduction zones around the circum-Pacific seismic belt^3^, even though some happen at shallow depth, notably in New Zealand^4–6^ and Costa Rica^7,8^. Slow-slip events at subduction zones worldwide often embody similar, characteristic features^9–13^. In particular, we observe small stress drops in the range 10–200 kPa, short recurrence times of a few months to a few years, and sometimes more regular periodicity than for earthquakes^14^. Some characteristics of slow-slip events can be explained within the context of rate-and-state friction^15,16^ with conditionally stable behavior^17–21^. Runaway frictional instabilities require a critical size for nucleation that depends on the frictional parameters and the effective confining pressure. For sufficiently small velocity-weakening asperities, only creep spontaneously occurs. Slow-slip events emerge spontaneously when the asperity size is commensurate to critical nucleation size^22^. As slow-slip events are now found close to universally at subduction zones^23–28^, it is unrealistic to expect the same combination of parameters across widely distant regions (Fig. 1A). Several authors developed and investigated other friction laws that regularize fast slip with a velocity-weakening to a velocity-strengthening transition^29–32^ or with dilatancy of the pore space^33,34^ that allows nucleation but resists fast slip. These models assume that slow-slip events represent brittle behavior and localization of deformation on the megathrust. Other authors investigated semi-brittle models for phyllicate-rich fault zones that include a frictional strength and the deformation of a mature fault gouge^35–37^, but their implications for slow slip are not known.

The location of slow slip around the continental Moho suggests a control by composition or rheology. The mantle wedge of subduction zones is filled with metamorphised upper mantle rocks in the form of antigorite-rich serpentinite (Fig. 1B), a high-pressure, low-temperature hydrous mineral characterized by a low activation energy for dislocation creep^38–40^. Laboratory experiments showed that serpentinite is velocity-strengthening at the low temperatures and pressures of the seismogenic zone, but velocity-weakening at and above 450 °C, at the pressure-temperature conditions of the corner of the mantle wedge, compatible with the occurrence of slow-slip events there^41^. Furthermore, laboratory friction experiments indicate that serpentine faults are characterized by a low healing rate and a large slip-weakening distance that promotes conditional stability, consistent with the slip mechanism of slow earthquakes^42^. The dominant flow mechanism of antigorite-rich serpentinite shows a transition from semi-brittle (localized) flow by strain localization to ductile (distributed) flow by intracrystalline deformation with increasing temperature from 300 °C to 500 °C at confining pressures from 1 to 2 GPa^43,44^. Serpentinite exhibits nonlinear plastic flow with an effective viscosity much lower than that of the major mantle-forming minerals^38^ allowing it to localize deformation. These laboratory results and field observations for exposed shear zones^45–47^ suggest that slow-slip events may be the result of mechanical instabilities that develop in a semi-brittle medium governed by both brittle and viscoelastic constitutive response.

Here, we present a model where, in addition to brittle rate-and-state friction, the strain and nonlinear rheology accommodated by a serpentinite layer are taken into account. We describe the semi-brittle constitutive model in Section 2. We then discuss the stability of semi-brittle fault zones in a simplified setting in Section 3. With this insight, we discuss the emergence and characteristics of slow-slip events in brittle and semi-brittle fault zones using earthquake cycle simulations performed in plane strain considering shear heating and thermal diffusion (Section 4). We find that fast ruptures can be damped out in a semi-brittle medium, resulting in the spontaneous generation of slow slip over a wider range of frictional parameters than for a brittle medium.

## The semi-brittle mechanical model

To model the semi-brittle fault zone, we first introduce a point-like spring-slider-dashpot system. This mechanical system consists of a spring-slider obeying rate-and-state friction in series with a viscous dashpot (Fig. 1B). The constitutive law is given by

1 $$\begin{array}{rcl}V & = & {V}_{{\rm{f}}}+{V}_{{\rm{d}}},\\ {V}_{{\rm{d}}} & = & 2{W}_{d}\dot{\varepsilon },\end{array}$$

where *V*_f_ is the velocity of the slider governed by rate-and-state friction, and *V*_d_ is the velocity of the viscous dashpot, which is controlled by a thermally-activated strain rate $\dot{\varepsilon }$. *W*_d_ is a characteristic width, taken as the width of the ductile shear zone near the fault, and is typically less than a few hundred meters^45,48^. The description of rate-and-state friction reads as

2 $$\begin{array}{rcl}\tau  & = & \mu \bar{\sigma }\\ \mu  & = & {\mu }_{0}+a\,\mathrm{log}\,\frac{{V}_{{\rm{f}}}}{{V}_{0}}+b\,\mathrm{log}\,\frac{{V}_{0}\theta }{L}\\ \dot{\theta } & = & 1-\frac{{V}_{{\rm{f}}}\theta }{L}.\end{array}$$

Here, *τ* is the fault shear stress, *μ* is the dynamic coefficient of friction, and $\bar{\sigma }=\sigma -p$ is the effective normal stress, with *p* the pore pressure. The friction coefficient depends on a state variable *θ*, whose evolution is given by an aging law $\dot{\theta }({V}_{{\rm{f}}},\theta )$. *V*_0_ and *L* are a reference slip rate and a characteristic weakening distance, respectively. For *V*_d_ = 0 we have a classical description of rate-and-state friction^15,16^, which reproduces many of the natural behaviors observed in real faults^49–54^. Such behaviors include unstable stick-slip cycles, stable creep, restrengthening following rapid slip, and rate-dependence. However, the classical description does not accommodate viscous effects. To account for this, we place a viscous dashpot in series with the spring-slider, and allow ${V}_{d}=2{W}_{d}\dot{\varepsilon }$ to be nonzero. The strain rate is given by a dislocation creep law^55,56^

3 $$\dot{\varepsilon }=A{\tau }^{n}\,\exp (-\frac{Q+pV}{RT}),$$

where *A* is a reference strain rate, *n* is the dislocation creep exponent, *Q* is the activation energy, *p* is confining pressure, *V* is the activation volume, *R* is the ideal gas constant, and *T* is temperature. For modeling purposes we use the empirically-determined creep parameters for serpentinite^38^. The serpentinite effective viscosity $\eta =\tau /\dot{\varepsilon }$ obeys an Arrhenius-style activation law with a low activation energy, and even at the 300–500 °C temperatures of subduction zones, viscous flow can be significant and play a nontrivial role in the dynamics of fault slip. It may be the case that once slip begins to accelerate due to frictional instabilities, viscous effects modulate the slip velocity.

The governing equation for the spring-slider-dashpot is given by

4 $$\tau =K(\delta -{V}_{{\rm{pl}}}t)-\frac{GV}{2{V}_{{\rm{s}}}}.$$

Here, *δ* is the total slip, with $\dot{\delta }=V={V}_{{\rm{f}}}+{V}_{{\rm{d}}}$. The spring stiffness is *K*. There is a driving plate rate *V*_pl_, which we can take to be the rate of plate convergence for a subduction zone, typically on the order of 10^−9^ m/s. The final term is the radiation damping approximation, used to account for inertial (body) terms in the equation of motion when modeling a finite fault. *G* is the shear modulus and *V*_s_ is the shear-wave speed. The constitutive laws (1–3) and the governing Equation (4) completely specify the system.

## Stability of semi-brittle fault zones

First, we consider the classical model of rate-and-state friction with *V*_d_ = 0 and ignoring the radiation damping term. Perturbing the equations about steady-state and linearizing yields a result for the critical spring stiffness^16^

5 $${K}_{{\rm{c}}}=\frac{\bar{\sigma }(b-a)}{L}.$$

If *K* > *K*_c_, then perturbations from steady-state are stable and the system shows a decaying oscillatory mode in phase space^57^. If *K* < *K*_c_, then perturbations from steady-state are unstable and the system’s oscillations grow without bound in phase space. One can observe a continuum of behaviors between slow-slip events^21^ and large elastodynamic ruptures indexed by the stiffness ratio *K*/*K*_c_, as also observed in laboratory experiments^58–60^. A larger ratio implies more stable and slower slip events, whereas a smaller ratio implies instability and fast slip. Alternating slow and fast slip events also occur close to the unit stiffness ratio^22^.

For the semi-brittle model of Section 2 with *V*_d_ ≠ 0, the stiffness becomes

6 $${K}_{{\rm{c}}}=\frac{\bar{\sigma }(b-a)}{L}\frac{{V}_{{\rm{f}},{\rm{s}}{\rm{s}}}}{{V}_{{\rm{f}},{\rm{s}}{\rm{s}}}+aC},$$

with *C* a positive constant given by

7 $$C=2{W}_{d}A{\bar{\sigma }}^{n}\,\exp (-\frac{Q+pV}{RT})\,n\,{\mu }_{{\rm{s}}{\rm{s}}}^{n-1}.$$

This result was obtained by performing a linear stability analysis for the spring-slider-dashpot in the same manner as for the spring-slider (see supplementary materials). The subscript label ss denotes the steady-state values for the system. Note that for the spring-slider, *V*_f,ss_ = *V*_pl_ and *C* = 0, so that (6) reduces to (5) in the limit of vanishing *V*_d_. The immediate significance of this result is that the critical stiffness is lowered in a partially viscous system. Thus, a wider range of the continuum of behaviors is occupied by stable, slow slip than for a purely rate-and-state frictional system. A set of frictional parameters that would have caused a fast rupture could instead result in a slow-slip event when viscous flow is activated.

## Semi-brittle models of slow-slip events

We investigate whether the constitutive properties of the semi-brittle medium can reproduce the long durations, small stress drops, and short recurrence times of slow-slip events. We assume that slow-slip events take place in a highly foliated antigorite-rich serpentinite shear zone that exhibits both brittle and ductile behavior (Fig. 1B). We assume that the brittle behavior is captured by rate-and-state friction, and that the viscoelastic flow obeys a nonlinear power-law rheology of the form (3). An important feature of the plastic flow of serpentinite is the thermal activation in the form of an Arrhenius law. Even though serpentinite has a small activation energy of *Q* = 13.3 kJ/mol^38^, the potentially high dynamic range of temperature due to shear heating during fault slip may play an important role near the fault^61^. While we did not consider shear heating in the linear stability analysis of Section 3 for the sake of simplicity, we do incorporate it in the present numerical model. In particular, substantial thermal weakening may occur in the ductile shear zone that may actually enhance the rupture. To test the importance of this effect in the dynamics of slow slip, we build a two-dimensional model of fault slip that includes shear heating and the diffusion of temperature away from the fault^62^

8 $$\dot{T}({\bf{x}})=k{{\rm{\nabla }}}^{2}T+\frac{\tau }{\rho c}[\frac{{V}_{f}}{2{W}_{f}}{\rm{\Omega }}(\frac{{\bf{x}}\,\cdot \,\hat{n}}{2{W}_{f}})+\frac{{V}_{d}}{2{W}_{d}}{\rm{\Omega }}(\frac{{\bf{x}}\,\cdot \,\hat{n}}{2{W}_{d}})]$$

where *T* is the deviation from the background temperature, *k* is the thermal diffusivity, *V*_*f*_ is the velocity of fault slip, *V*_*d*_ is the integrated velocity across the shear zone due to ductile strain, *ρc* is the specific heat per unit volume of the fault gouge, *W*_*f*_ is the gouge width, *W*_*d*_ is the width of the shear zone, and $\hat{n}$ is the fault normal. Ω(*y*) = 1 for *y* in [−1/2, 1/2] and 0 elsewhere. We ignore the thermal pressurization of pore fluids^63^ as they become important for large slip typical of great earthquakes^64^.

**Figure 2:**
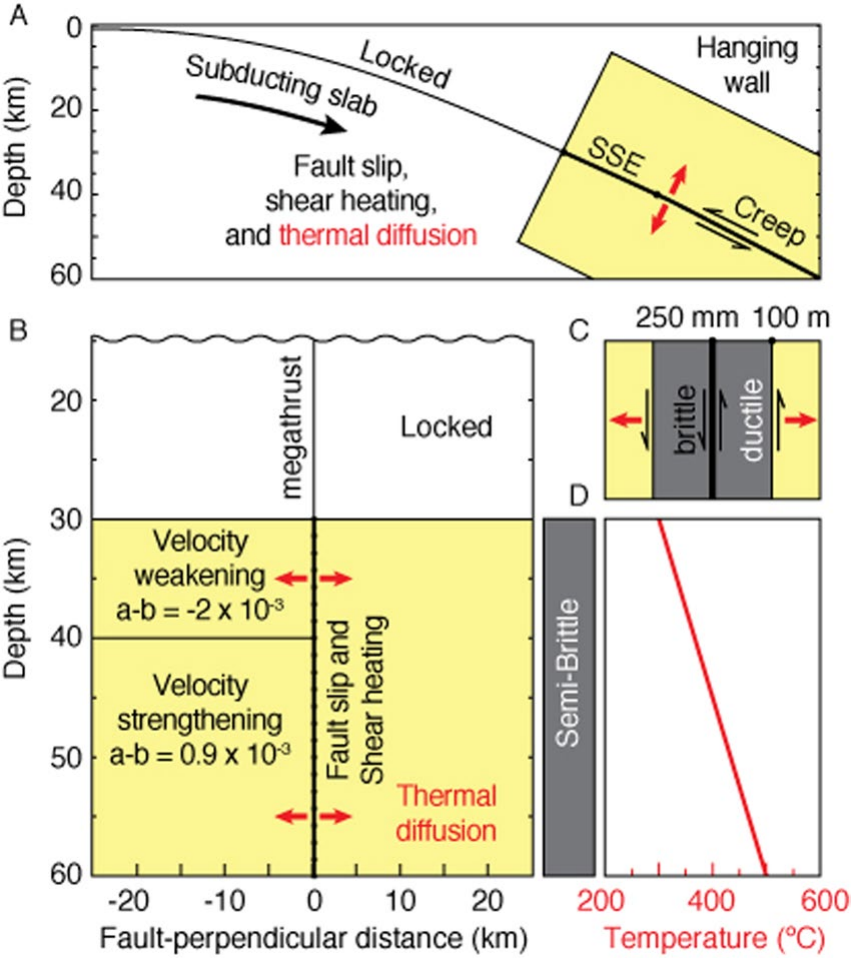
Schematic of the numerical model. (**A**) We evaluate the dynamics of semi-brittle deformation from 30 to 60 km depth. The shallow portion of the megathrust is assumed locked at all times during the simulation. We mesh the domain surrounding the fault (yellow region) to evaluate shear heating and thermal diffusion. (**B**) The mesh is rectilinear in a reference system aligned with the fault orientation. The non-uniform mesh is finer near the fault and larger in the far field. Diffusion is allowed in the fault-parallel and the fault-perpendicular directions. The fault is velocity-weakening from 30 to 40 km depth. (**C**) Each fault patch consists of a brittle surface and a surrounding ductile shear zone. The two modes of deformation accommodate subduction of the oceanic plate. (**D**) We assume a linear geothermal gradient as initial condition of the thermomechanical model.

We consider a two-dimensional finite-element thermomechanical model of a subduction zone megathrust with a one-dimensional fault surface consisting of *N* = 200 spring-slider-dashpot elements at depths of 30–60 km (Fig. 2). We adopt the same modeling strategy of ^65^ by simulating slow-slip events during the interseismic period. Accordingly, we assume that the updip portion of the model remains locked at all times during the simulation. The fault elements are mechanically coupled to one another with an *N* by *N* full stiffness matrix *K* following the integral method^66^. Instead of solving for the inertial contribution directly, we incorporate the radiation damping approximation at high slip velocities^67^. To allow for the possibility of shear heating and dynamic thermal effects, we take temperature to be a dynamic variable. The simulation method involves a set of coupled first-order ordinary differential equations evolved forward in time, and allows for temperature diffusion using finite differencing. For the time stepping, we employ the four/fifth-order Runge-Kutta method with adaptive time steps^68^. This method allows us to accurately resolve the rapid dynamics of fault slip and the long periods of quiescence during the interseismic period. As the time steps are adapted automatically, this allows us to capture all the instabilities, slow or fast, that emerge spontaneously.

We assume a small effective normal stress of $\bar{\sigma }=6.25$ MPa, compatible with the small stress drop of slow-slip events and their sensitivity to small external perturbations^69–73^. The low effective normal stress may be caused by high pore pressures associated with the dehydration of the downgoing slab^43,74,75^. Note, however, that our model does not include dynamic dehydration. We choose a static friction coefficient of *μ*_0_ = 0.2 compatible with wet serpentinite^48,76^. For the velocity-weakening segment, we choose a depth range of 30–40 km and *a* − *b* = −2 × 10^−3^. We consider 40–50 km depths to be velocity-strengthening at *a* − *b* = 0.9 × 10^−3^. A fault half-width of *W*_*f*_ = 250 mm is used to generate frictional shear heating^63^. We assume a width of 100 m for the extent of the viscous shear zone, compatible with outcrops of serpentinite breccia^48^. Inside the shear zone, the strain is assumed uniform in the fault-perpendicular direction. We model the megathrust dynamics with fault patches (and their surrounding ductile zone) of 150 m downdip width that is largely sufficient to resolve the critical nucleation size of h^*^ = 8.4 km. We use a linear temperature profile in the range 300–500 °C over the modeled depths as an initial condition. This profile is representative of temperatures found at subduction zones that exhibit long-term SSEs, such as Nankai, Mexico, and Hikurangi^77^.

For the thermal diffusion in the fault perpendicular direction, we adopt a non-uniform mesh that extends symmetrically ±25 km away from the fault, in which the few samples closest to the fault have sub-meter thickness (25 cm) on both sides. One-sided finite differencing is used as a boundary condition at the lateral edges (±25 km) of the mesh, but this is far enough away from the fault such that the specific details of the condition does not affect the fault behavior significantly. Since the timesteps are adapted so that integration is always carried out within a specified tolerance, difficulties associated with the Courant-Friedrichs-Lewy (CFL) condition for explicit finite differencing can be circumvented. The model parameters are summarized in Table 1.

**Table 1:** Model parameters for lithosphere-asthenosphere dynamics.

	Parameter	Symbol	Value
Frictional Parameters	Shear modulus	*G*	30 GPa
	Effective normal stress	$\bar{\sigma }$	6.25 MPa
	Static friction coefficient	*μ*_0_	0.2
	Direct effect parameter	*a*	1 × 10^−3^
	State evolution parameter	*b*	1 × 10^−4^ (velocity-strengthening)
			3 × 10^−3^ (velocity-weakening)
	Characteristic weakening distance	*L*	3.5 mm
	Plate rate	*V*_*pl*_	10^−9^ m/s
	Reference slip velocity	*V*_0_	10^−6^ m/s
	Shear wave speed	*V*_*s*_	3 × 10^3^ m/s
Rheological Parameters	Reference strain rate	*A*	3.879 × 10^−9^ MPa^−*n*^ s^−1^
	Power-law exponent	*n*	3.0
	Activation energy	*Q*	13.3 kJ/mol
	Activation volume	Ω	3.16 × 10^−6^ m^3^/mol
	Density	*ρ*	3300 kg/m^3^
Thermal Parameters	Specific heat per unit volume	*ρc*	2.7 MPa/K
	Thermal diffusivity	*k*	8 × 10^−7^ m/s^2^
	Gouge width	*W*_*f*_	250 mm
	Shear zone width	*W*_*s*_	100 m

Rheological parameters used from laboratory studies for wet dislocation creep conditions in olivine^38^.

**Figure 3:**
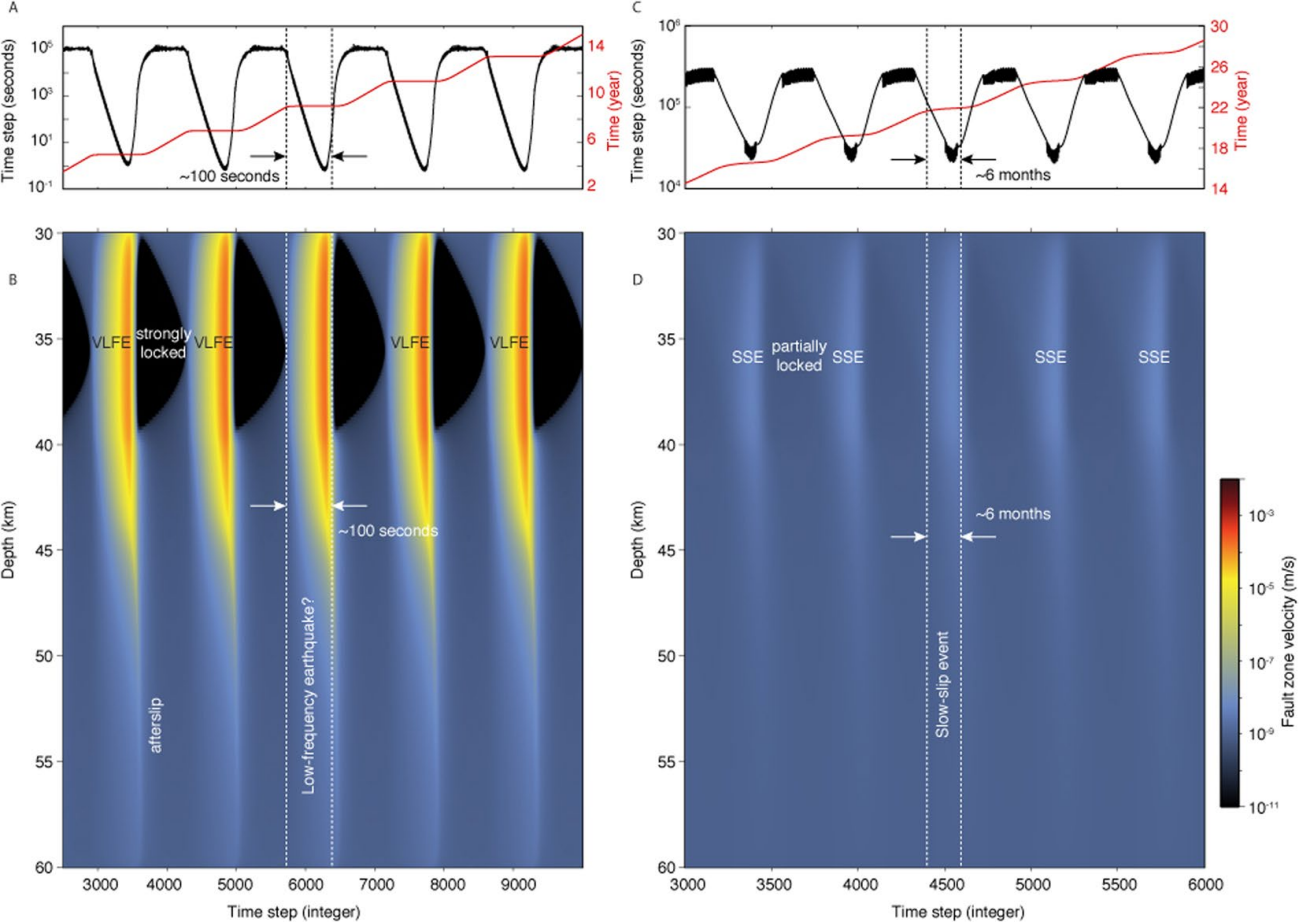
Comparison of the dynamics of slow slip in brittle (left) and semi-brittle (right) media. The left column (**A**,**B**) depicts fast ruptures in the limit of no viscous shear, *V*_d_ → 0, corresponding to the end-member case for a peridotite mantle wedge with a high activation energy, or a cold geotherm. The right column (**C**,**D**) depicts slow-slip events obtained using a low-activation energy serpentinite rheology. The frictional parameters used for both models are identical. (**A**,**C**) Time steps (black profile) and cumulative time (red profile) employed in the numerical model, used here as a proxy for the frequency content of the source. (**B**,**D**) Evolution of the slip velocity as a function of computational time steps. The brittle and semi-brittle models both exhibit dominantly aseismic slip considering the criterion (9). The brittle model has a higher frequency content than the semi-brittle model. The brittle model has energy up to 1 Hz. This range is representative of very low frequency earthquake (VLFE) events. The semi-brittle model shows longer-duration aseismic events with no energy above 0.1 mHz, similar to slow-slip events (SSEs). Each SSE is separated by a period of partial locking.

**Figure 4:**
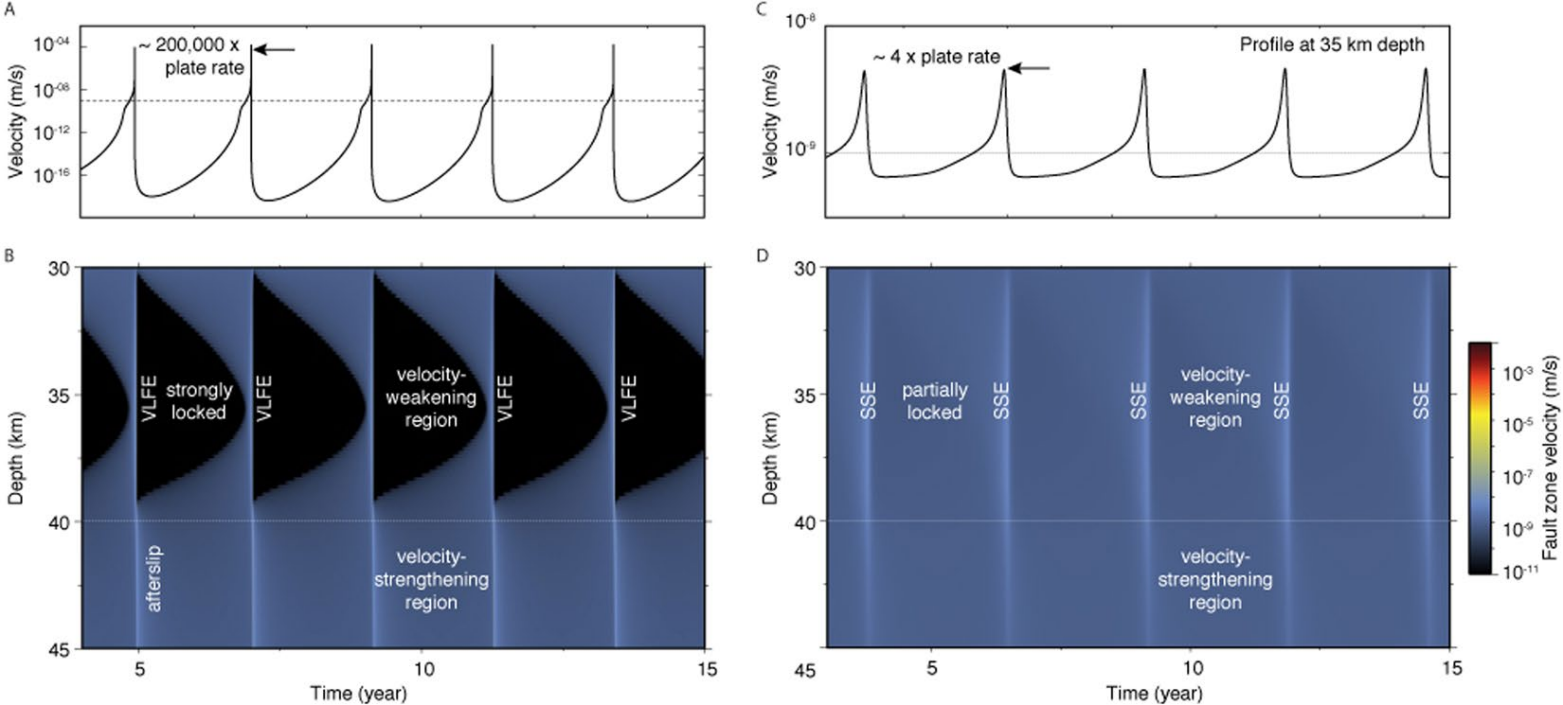
Comparison of the dynamics of slow slip in brittle (left) and semi-brittle (right) media (same as Fig. 3 but with physical time). (**A**) Slip velocity as a function of time at 35 km depth, at the center of the velocity-weakening region for the brittle model. (**B**) Slip velocity for all fault patches in the depth range 30–45 km for the brittle model. (**C**) Slip velocity at the center of the velocity-weakening region for the semi-brittle model. (**D**) Slip velocity in the semi-brittle model for all patches between 30 and 45 km depth. The brittle model exhibits fast, impulsive slip, while the semi-brittle model peak velocity is only about four times the background loading rate and more emergent.

We compare the dynamics of fault slip for the brittle and semi-brittle cases. Brittle deformation represents the end-member for a peridotite mantle wedge with a high activation energy, or equivalently a cold geotherm, such as that at the Japan Trench. In both cases viscoelastic flow is negligible. In the brittle and semi-brittle models, the frictional properties are identical. We evaluate the resulting dynamics of fault slip for 30 years, representing about 15 events with recurrence times between 2 and 2.5 years (Figs 3 and 4). The relative importance of radiated energy is controlled by the peak slip velocity during a slip event. Slip events with peak velocity below the threshold^78,79^

9 $${V}_{{\rm{th}}}=2{V}_{s}\frac{a\bar{\sigma }}{G}$$

emanate little radiation and can be considered dominantly aseismic. For the parameters in this study, *V*_th_ = 1.25 × 10^−3^ m/s. Peak slip rates for both the brittle and semi-brittle models are less than this threshold. In the case of brittle deformation (left panel in Fig. 4), we obtain fast slip events with peak velocities of 10^−4^ m/s, corresponding to ~200, 000 times the background plate rate. Even though these events are dominantly aseismic considering the criterion (9), they are impulsive and last about ~100 seconds. Using the simulation time steps as a proxy for frequency content, we infer that the source of these events possess energy around 1 Hz, making them detectable by standard seismological instruments. They may be considered the source of very low frequency earthquakes (VLFEs) that emanate seismic energy in narrow, low frequency bands compared to normal earthquakes^1^. When the presence of low-activation energy serpentinite is taken into account (right panel in Figs 3 and 4), the slip events are more emergent and last of the order of 6 months with a peak velocity of about four times the background plate rate. The computational time steps are all higher than 2 × 10^4^ s, indicating a frequency content lower than detectable with broadband seismometers (~0.1 mHz), making these events practically aseismic.

**Figure 5:**
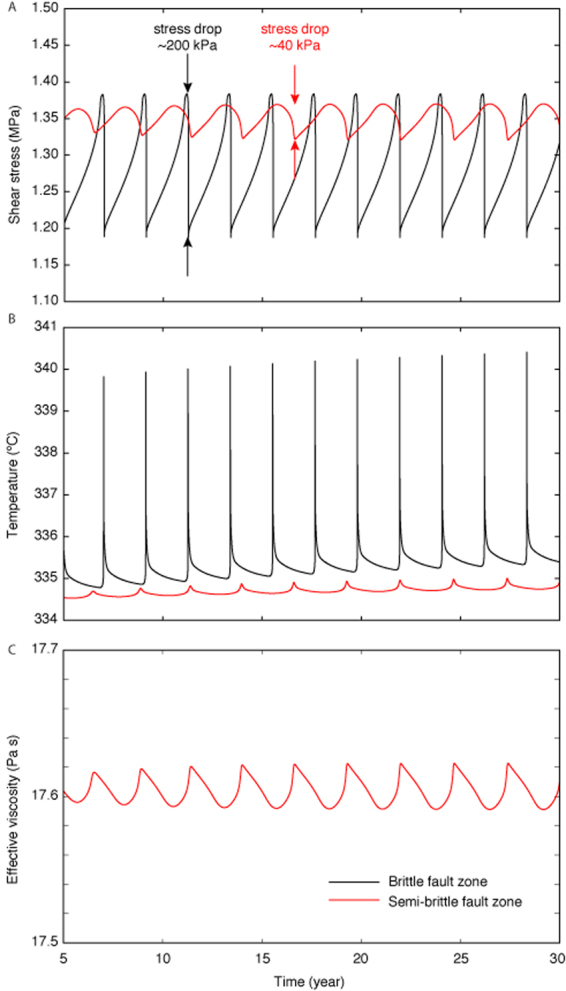
Comparison between the source properties of the brittle and semi-brittle models during multiple cycles. (**A**) Evolution of shear stress. Both models exhibit small stress drops, but with ~40 kPa, the semi-brittle model is closer to the observed average stress drop of slow-slip events at subduction zones. (**B**) Evolution of fault zone temperature due to shear heating and thermal diffusion. The dynamic range of temperature in the brittle and semi-brittle models is modest, precluding strong thermal weakening. (**C**) Evolution of the effective viscosity of the power-law flow due to the combined effect of temperature and stress variations. The changes of temperature do not create strong reductions of effective viscosity due to the low activation energy of serpentinite and the limited cumulative slip during slow-slip events.

The stress drops for both models fall within the range of observations on natural faults (Fig. 5), but while the stress drops for the brittle model are on the high range (~200 kPa), the ones for the semi-brittle model (~50 kPa) are more representative of the average of slow-slip event observations across subduction zones.

Following our concern about the role of shear heating, we evaluate the dynamics of temperature and resulting effective viscosities in the fault zones for both models (Fig. 5). The temperature increases more during mechanical instabilities in the brittle model due to the higher slip velocities and the narrower distribution of shear (*W*_*f*_ < *W*_*d*_) in this case. In both cases, the dynamic range is less than a few degrees. In the semi-brittle model, the effective viscosity varies within 0.1% of the average background value *η*_effective_ = 4 × 10^17^ Pa s (Fig. 5C). The effective viscosity is also modulated in the brittle case, but the high-activation-energy fault zone has an effective viscosity many orders of magnitude higher at these temperature and pressure conditions. These results indicate that while shear heating does affect viscosity in the fault zone, the variations are not sufficient to melt the fault or produce significant additional weakening due to thermally activated viscosity during slow-slip events. However, our results do not preclude a more important role of shear heating for other regimes and faster slip velocities^80–85^. We find that the initial temperature profile plays a more important role. The qualitative features of our solution are robust to within about ±30 °C of the initial temperature condition. The model becomes brittle for a geotherm more than 30 °C colder, and ductile for a geotherm more than 30 °C warmer. Our model may explain the presence of long-term SSEs at the Nankai, Mexico, and Hikurangi subduction zones, where temperatures in the range 300–500 °C are found at the mantle wedge corner. Our model may also explain the absence of long-term SSEs at the Japan Trench where the mantle wedge is cold, and at the Northern Cascadia subduction zone where the 300–500 °C region is relatively narrow^77^.

Slow-slip events at subduction zones exhibit complex patterns of rupture propagation highlighted by rapid tremor reversals^23,86–88^. Our two-dimensional numerical models are limited to one direction of slip, but the rupture velocity can be related to the dynamic stress drop and the peak slip velocity^89,90^

10 $${V}_{r}\approx {V}_{p}\frac{1}{1-\nu }\frac{G}{{\rm{\Delta }}{\tau }_{{\rm{dyn}}}}.$$

With a peak velocity *V*_*p*_ = 2 × 10^−9^ m/s, and a dynamic stress drop of Δ*τ*_dyn_ = 50 kPa (Figs 4 and 5), we estimate the rupture velocity *V*_*r*_ = 0.3 km/day, commensurate with, but on the low range of, along-strike propagation velocities^12,91,92^. An important shortcoming of our two-dimensional model is that we cannot discuss the faster tremor propagation velocity in the updip direction^92,93^.

## Discussion and conclusion

The simulated events in the semi-brittle model reproduce many characteristic features of natural slow-slip events. In particular, we note a recurrence time of about two years, a stress drop of about 50 kPa, a slip rate just a few multiples of plate rate, and a long duration. While slow-slip events can be obtained in purely brittle models of slip evolution within the rate-and-state framework, our study shows that viscoelastic flow in a narrow shear zone centered about the fault can regularize fast slip, thereby permitting slow slip for a wider range of frictional parameters. If the semi-brittle constitutive behavior is associated with a serpentinized plate interface below the forearc Moho, it may explain the near-universal presence of slow-slip events around subduction zones at these depths.

We note an important difference between our semi-brittle model and others. While those models with a transition from velocity-weakening to velocity-strengthening at high slip speeds systematically damp fast slip, our semi-brittle model produces slow-slip events only for a given range of frictional and rheological parameters (see Section 3) and may produce fast elasto-dynamic ruptures under other conditions. In particular, ruptures that propagated in the seismogenic zone may continue to propagate in the semi-brittle region.

These results highlight the importance of rheological behavior for fault dynamics and seismic hazards. While serpentinite is thought to inhibit the nucleation of seismic rupture^43^, it may be responsible for the dynamic stabilization of fault slip in the mantle wedge corner, as also inferred from laboratory studies^41^. While slow-slip events are thought to arrest ruptures^94^, we note that antigorite shear zones may still play an important role in controlling the propagation of earthquake ruptures by the formation of partially amorphized material due to a combination of flash weakening and thermal pressurization^95^. The degree of metamorphism of serpentinite within the mantle wedge corner may also be responsible for the rheological separation of slow-slip events and episodic tremor and slip^2,77^, ‘perhaps corresponding to various degrees of dominance of the ductile behavior’.

### Data availability

The datasets generated during and/or analysed during the current study are available from the corresponding author on reasonable request.

## Electronic supplementary material

Supplementary Information
