# Supplementary material for: Slow-slip events in semi-brittle serpentinite fault zones
Source: Sci Rep. 2018 Apr 18;8:6181. doi: 10.1038/s41598-018-24637-z (PMC5906616; doi:10.1038/s41598-018-24637-z)
Supplement: Supplementary file 1 — Supplementary Information [file 41598_2018_24637_MOESM1_ESM.pdf]

Supplementary Materials to "Slow-slip events in semi-brittle serpentinite fault zones"

A. Goswami & S. Barbot

Linear stability analysis

We describe the details of the linear stability analysis of the spring-slider-dashpot constitutive properties for a point-like element. At equilibrium, and ignoring radiation damping, we have

$$K(\delta - V_{\rm pl}t) = \bar{\sigma}\left[\mu_0 + a\ln\left(\frac{V_{f,ss}}{V_0}\right) + b\ln\left(\frac{V_0\theta_{,ss}}{L}\right)\right] \ , \tag{11}$$

where we have defined

$$\begin{aligned} V_f &= V_{f,ss} + \Delta V_f \\ \theta &= \theta_{,ss} + \Delta\theta \end{aligned} \tag{12}$$

with $\theta_{,ss} = L/V_{f,ss}$, and the subscript $,ss$ refers to steady state. We now derive the perturbation equation by differentiating about steady state, as follows

$$\begin{aligned} \bar{\sigma}\Bigg[\mu_0 + a\ln\left(\frac{V_{f,ss}}{V_0}\right) + a\frac{\partial}{\partial V_f}\ln\left(\frac{V_f}{V_0}\right)\Bigg|_{V_{f,ss}} \\ + b\ln\left(\frac{V_0\theta_{,ss}}{L}\right) + b\frac{\partial}{\partial\theta}\ln\left(\frac{V_0\theta}{L}\right)\Bigg|_{\theta_{,ss}}\Bigg] = K(\delta + \Delta\delta - V_{\rm pl}t) \end{aligned} \tag{13}$$

Making use of (11), the above expression simplifies to

$$\bar{\sigma}\left[a\frac{\partial}{\partial V_f}\ln\left(\frac{V_f}{V_0}\right)\Bigg|_{V_{f,ss}} + b\frac{\partial}{\partial\theta}\ln\left(\frac{V_0\theta}{L}\right)\Bigg|_{\theta_{,ss}}\right] = K\,\Delta\delta \ . \tag{14}$$

Expanding the derivatives, we obtain

$$\bar{\sigma}\left(a\frac{\Delta V_f}{V_{f,ss}} + b\frac{\Delta\theta}{\theta_{,ss}}\right) = K\,\Delta\delta \ . \tag{15}$$

With $\theta_{,ss} = L/V_{f,ss}$, taking the time derivative, and using $\Delta\dot{\delta} = \Delta V_f + \Delta V_d$, we obtain

$$\bar{\sigma}\left(a\frac{\Delta\dot{V}_f}{V_{f,ss}} + b\frac{V_{f,ss}}{L}\Delta\dot{\theta}\right) = K\,\left(\Delta V_f + \Delta V_d\right) \ . \tag{16}$$

In addition, the perturbation of the aging-law gives

$$\Delta\dot{\theta} = -\frac{\Delta V_f}{V_{f,ss}} - \frac{V_{f,ss}}{L}\Delta\theta \ . \tag{17}$$

Similarly expanding the constitutive law (3) for the viscous dashpot about steady state gives

$$\Delta V_d = C\left(a\frac{\Delta V_f}{V_{f,ss}} + b\frac{V_{f,ss}}{L}\Delta\theta\right) \tag{18}$$

where C is a positive constant given by

$$C = 2W_d A\bar{\sigma}^n \exp\left(-\frac{Q}{RT}\right) n\mu_{ss}^{n-1}. \tag{19}$$

For simplicity we have replaced $Q+pV$ with Q. Combining (16), (17), and (18), we get

$$\bar{\sigma}\left(a\frac{\Delta\dot{V}_f}{V_{f,ss}} + b\frac{V_{f,ss}}{L}\Delta\dot{\theta}\right) = K\left(1+\frac{aC}{V_{f,ss}}\right)\Delta V_f + KbC\frac{V_{f,ss}}{L}\Delta\theta \ . \tag{20}$$

The steady-state velocity of the slider is the fixed point of the following transcendental equation

$$V_{f,ss} + 2WA\bar{\sigma}^n \exp\left(-\frac{Q}{RT}\right)\left[\mu_0 + (a-b)\ln\left(\frac{V_{f,ss}}{V_0}\right)\right]^n = V_{\mathrm{pl}} \ , \tag{21}$$

which can be solved numerically for $V_{f,ss}$. In turn, we have $V_{d,ss} = V_{\mathrm{pl}} - V_{f,ss}$. The set of linear ordinary equations for can be written in matrix form

$$\begin{pmatrix}\Delta\dot{V}_f\\ \Delta\dot{\theta}\end{pmatrix} = \begin{pmatrix} A & B\\ -\frac{1}{V_{f,ss}} & -\frac{V_{f,ss}}{L}\end{pmatrix}\begin{pmatrix}\Delta V_f\\ \Delta\theta\end{pmatrix} \ , \tag{22}$$

where

$$\begin{aligned} A &= \frac{bV_{f,ss}}{aL} + \frac{K}{\bar{\sigma}}\left(C + \frac{V_{f,ss}}{a}\right)\\ B &= \frac{b}{aL^2}V_{f,ss}^3 + K\frac{bC}{a\bar{\sigma}}\frac{V_{f,ss}^2}{L}\end{aligned} \tag{23}$$

To establish the stability of the system of equations, we consider the eigenvalues of the 2 by 2 stability matrix, which are

$$\frac{1}{2aL^2V_{f,ss}\sigma}\left(KL^2V_{f,ss}(aC+V_{f,ss}) + (b-a)LV_{f,ss}^2 \pm LV_{f,ss}\sqrt{\text{radicand}}\right) \tag{24}$$

where

$$\text{radicand} = 4aKLV_{f,ss}((a-b)C+V_{f,ss})\sigma + (aCKL + KLV_{f,ss} - (a-b)V_{f,ss}\sigma)^2 \tag{25}$$

The stability is determined by the sign of the eigenvalue's real part. If the radicand is negative for a given set of parameters, then the real part is negative whenever

$$K > \frac{\sigma(b-a)}{L} \frac{V_{f,ss}}{aC + V_{f,ss}} \tag{26}$$

This equation holds for positive values of the stiffness K. For small, nonzero C, this result is merely a perturbation of the analysis presented in[97].

Numerical solver

The equations that specify our system are specified in Section 2. We solve the dynamic equilibrium using a 4/5th order Runge-Kutta method where the dynamic variables are recast to write the system in the classic form

$$\dot{\mathbf{y}} = f(\mathbf{y}, t) \ , \tag{27}$$

where $\mathbf{y}$ is the state vector

$$\mathbf{y} = \left(\delta, V_f, V_d, \tau, \theta, T\right)^T \tag{28}$$

The variables are defined in the main text. The time derivative of the state variable and temperature are readily provided by the aging law (2) and the diffusion law (8), respectively. Below, we obtain time derivatives of the remaining dynamic variables. From (2), we have

$$\dot{\tau} = a\bar{\sigma}\frac{\dot{V}_f}{V_f} + b\bar{\sigma}\frac{\dot{\theta}}{\theta} \ . \tag{29}$$

From (3), we have

$$\begin{aligned} \dot{V}_d &= 2WA\tau^n \exp\left(-\frac{Q}{RT}\right)\left(n\frac{\dot{\tau}}{\tau} + \frac{Q}{RT}\frac{\dot{T}}{T}\right) \\ &= V_d\left(n\frac{\dot{\tau}}{\tau} + \frac{Q}{RT}\frac{\dot{T}}{T}\right) \ . \end{aligned} \tag{30}$$

From (1), (4), and $\dot{\delta} = V$, we have

$$\dot{\tau} = K\left(V_f + V_d - V_{pl}\right) - \frac{G}{2V_s}(\dot{V}_f + \dot{V}_d) \ . \tag{31}$$

Eqs. (29)-(31) form a system of three linear equations in the unknowns $\dot{\tau}, \dot{V}_f, \dot{V}_d$. Solving, we obtain

$$\frac{\dot{V}_f}{V_f} = \frac{2V_s\tau\, RT^2(K(V_d + V_f - V_{pl}) - b\sigma\dot{\phi}) - GV_d\left(bn\sigma RT^2\dot{\phi} + Q\tau\dot{T}\right)}{RT^2(a\sigma(2V_s\tau + GnV_d) + G\tau V_f)} \ , \tag{32}$$

and

$$\frac{\dot{V}_d}{V_d} = \frac{2ac\sigma \left(KnRT^2(V_d + V_f - V_{pl}) + Q\tau\dot{T} \right) + GV_f \left(bn\sigma RT^2\dot{\phi} + Q\tau\dot{T} \right)}{RT^2(a\sigma(2V_s\tau + GnV_d) + G\tau V_f)} , \tag{33}$$

where $\dot{\phi} \equiv \dot{\theta}/\theta$. After obtaining $\dot{V}_f$ and $\dot{V}_d$, $\dot{\tau}$ may be obtained from any of (29)-(31).
